# Designing nutritionally improved waffles containing glycerol monostearate based oleogel and aquafaba

**DOI:** 10.1002/jsfa.70717

**Published:** 2026-05-15

**Authors:** Cristian Szekely, Andreea Pușcaș, Andruța Elena Mureșan, Vlad Mureșan

**Affiliations:** ^1^ Food Engineering Department, Faculty of Food Science and Technology University of Agricultural Sciences and Veterinary Medicine Cluj‐Napoca Cluj‐Napoca Romania

**Keywords:** aquafaba, concentration, oleogel, sustainability, texture

## Abstract

**BACKGROUND:**

Plant‐based diets are considered healthier and more sustainable, while solutions are sought to replace saturated fats in foods products. Aquafaba (AF) from chickpea cans was foamed to formulate waffles. AF was concentrated at three dry matter contents. Gluten‐free oat flour and an oleogel (OG) containing 50 g kg^−1^ glycerol monostearate were also used as ingredients. Three factors (AF concentration, whipping time and type of fat) were varied on two levels to obtain the samples.

**RESULTS:**

Fourier transform infrared analysis revealed protein specific peaks that are responsible for the foaming properties of AF. Concentrated AF had a gel‐like behaviour (*G*′ > *G*′′), caused by the denaturation of proteins. AF21 was significantly stronger (*G*′ = 89.22 ± 3.17 Pa) compared to AF14 (*G*′ = 3.81 ± 0.3 Pa). Dough samples with AF7 and OG had a significantly higher hardness at the same whipping time compared to their oil counterparts (e.g. 10.78 ± 0.16 N for sample 6 *versus* 4.81 ± 0.34 N for 8). Waffles’ springiness was low due to the gluten‐free oat flour. A positive correlation (*r* = 0.743) was found between protein and hardness. One formulation was chosen for the sensory evaluation based on the principal component analysis. Principal component (PC) 1 accounted for 60.2% of the variance and PC 2 explained 24.4%. All samples had low hedonic scores and the most preferred sample was the control with wheat flour.

**CONCLUSION:**

This study demonstrates that AF concentration and the usage of OG influence the characteristics of waffles. © 2026 The Author(s). *Journal of the Science of Food and Agriculture* published by John Wiley & Sons Ltd on behalf of Society of Chemical Industry.

## INTRODUCTION

Sustainability refers to systems and processes that can function and persist on their own for a long period of time,^1^ and so, in today's world, even if economic growth has brought unprecedented benefits to humanity, its sustainable character has been questioned.^2^ The food industry plays an essential role for sustainability,^3^ with a positive emphasis on plants' role.^4^ A major global problem nowadays is hunger, which can be reduced by reducing food waste.^5^

A plant based diet can lower the risk of cardiovascular disease and has a sustainable environmental impact.^6^ Legumes are plants with a high protein content and, when processed in a canned form, they contain a liquid called ‘aquafaba’^7^ that can be used as an ingredient in certain culinary dishes,^5^ although often it is thrown away. Aquafaba (AF) can be used as an egg replacer in vegan recipes because of its techno‐functional properties such as foaming, emulsifying and gelling capacities,^8^ depending on its chemical composition,^9^ and it has already been explored in the formulation of various bakery or confectionary products such as cupcakes,^10^ gluten free bread,^11^ macarons^12^ and sponge cake,^13^ but, to our knowledge, not in the production of waffles.

Solid fats have superior techno‐functional properties related to food texture,^14^ although they contain mainly saturated fatty acids, which have been associated with negative health effects, such as cardiovascular disease and type 2 diabetes.^15^ One of the challenges of food manufacturers is to reduce the total saturated fat content of foods without altering their textural properties. In this regard, oleogels might be potentially used. Oleogels are semisolid lipid systems formed by turning a liquid oil into a solid‐like fat using oleogelators, thus having functional properties similar to saturated fats, but with a nutritional value closer to that of a liquid oil.^16^ Oleogelators (e.g. monoglycerides) entrap the oil into a three dimensional network and the applicability of oleogels has been successfully studied in food products such as chocolate, sausages and cakes.^15^

Waffles are products that are appreciated for their taste and texture,^17^ but they contain animal‐origin ingredients, such as eggs and high amounts of butter, which makes them unsuitable for vegan consumers and for those who prefer a low saturated fat diet.

Considering the information outlined above, the present study aimed to reformulate a conventional waffle recipe using foamed AF as an egg substitute and an oleogel (OG) formed with glycerol monostearate (GM) and sunflower oil to replace the butter. Additionally, gluten‐free oat flour (OF) was used to address the needs of people suffering from coeliac disease. The objectives were focused on studying the AF properties as influenced by the dry matter and to assess its techno‐functionality. With respect to the waffles, an optimal recipe was established via prior try‐outs and the present study aimed further to enhance the structural properties of the waffles using a two‐level, three‐factorial experimental design and to explore as a result the main physico‐chemical and mechanical parameters of the waffles. Also, a hedonic test and a preference test were performed to establish the acceptance of the nutritionally improved waffle, containing the ingredients AF, OG or OF.

## MATERIALS AND METHODS

### Materials

For the purpose of this study, ‘Freshona’ (Lidl, Stiftung & Co.KG, Neckarsulm, Germany) chickpea cans were used, which contain, according to the label, only chickpeas and water. All‐purpose wheat flour (Goodmills, Pantelimon, Romania) was used for the control waffles and gluten‐free oat flour (GymBeam, Berlin, Germany) for the reformulated waffles. Crystal sugar was manufactured by Agrana Romania S.R.L. (Bucharest, Romania) and sunflower oil by EXPUR S.A. (Bucharest, Romania). To obtain the oleogels, glycerol monostearate (ThermoFisher GmbH, Kandel, Germany) was used as oleogelator. The reagents used were of analytical grade.

### Experiments on AF

#### Refractive index determination and concentrating the AF

AF was first separated from the chickpea beans using a sieve and an Abbe refractometer was used to determine its refractive index. The AF concentration was then doubled and tripled, respectively, by evaporation at 102 °C on a hot plate, and this was used for the rest of the experiments.

#### Fourier transform infrared (FTIR) spectrometry

The FTIR spectra of the AF samples were recorded at a wavenumber range of 4000–450 cm^−1^ using Agilent Cary360FTIR (Agilent Technologies, Santa Clara, CA, USA). A droplet of sample was used to conduct the analysis at 25 °C, and the spectra were analysed using OriginPro 2024 (OriginLab Corp., Northampton, MA, USA).

#### Rheological measurements

An amplitude sweep test (0.01–100%) was performed using an Anton Paar MCR302 (Anton Paar, Graz, Austria) rheometer at 25 °C. The rheometer was equipped with a parallel plate geometry (PP50) and connected to a Julabo water bath (JULABO GmbH, Seelbach, Germany). The gap was set at 1 mm and the linear viscoelastic region of the samples was measured.

#### Whipping time determination

To determine the whipping time, 400 g kg^−1^ of sample was used for the experiments. AF was whipped in Berzelius beakers (diameter 50 mm; height 125 mm) using an electric mixer (HR1617/00; 650 W; Philips, Eindhoven, The Netherlands) equipped with a whisk attachment. The mixer was set on ‘turbo’ mode and after each minute of whipping the height of the foam was measured using a ruler. The whipping was conducted until overrun, when the foam started to collapse (i.e. its height started to decrease) and the optimal whipping time was considered the one for which the maximum height was recorded.

#### Foaming capacity determination

For this determination, AF was poured into plastic lids and weighed. Then, the same lids were used to weigh the foams obtained from AF whipped until their optimal time. The samples were handled such that their surface was perfectly aligned with the edge of the lid, without any voids present. The foaming capacity was calculated according to:

(1) $$F_{c}=\frac{w_{1}-w_{2}}{w_{1}}\times 100\;\left(\%\right)$$

where *F*_*c*_ is the foaming capacity, *w*_1_ is the weight before whipping (g) and *w*_2_ is the weight after whipping (g).

#### Serum loss

To assess the stability of the foams, the serum loss was investigated, defined as the quantity of the liquid that drained from the foams placed at the refrigeration temperature. In that regard, foams were whipped until their optimal time as previously determined and 50 g kg^−1^ of sample was collected into glass funnels with filter paper and then placed in Erlenmeyer glasses for 3 days at 4 °C to collect the drained liquid. Afterwards, the liquid was weighed and the foam stability was determined as the serum loss reported as a percentage to the initial weight of the foam.

### Experiments on waffles

#### Oleogel preparation

**Figure 1:**
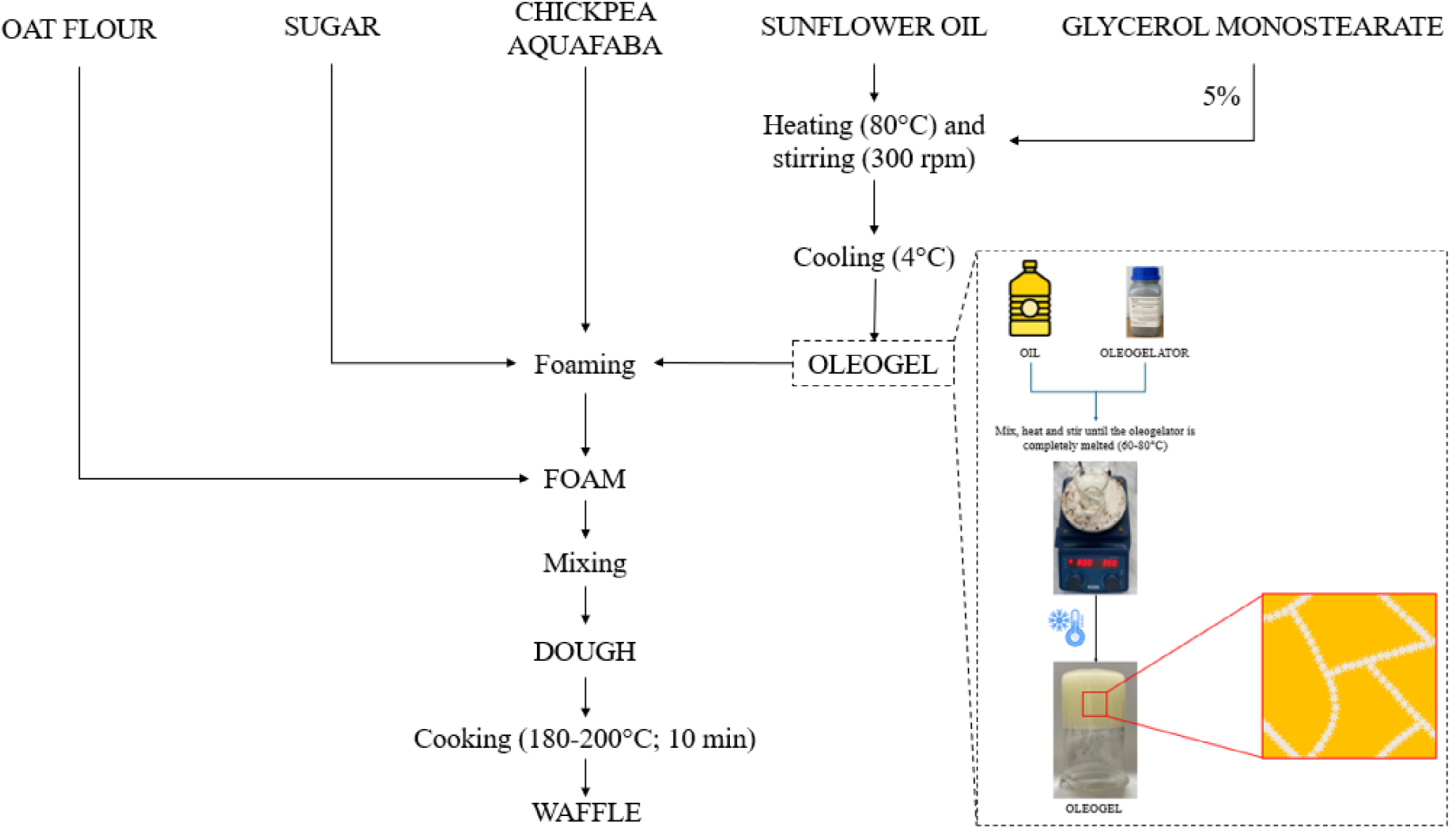
The production flow of the waffles and oleogel development.

The OG was obtained using sunflower oil and glycerol monostearate (Fig. 1). First, 50 g kg^−1^ glycerol monostearate was added to the oil, followed by heating the mixture at 80 °C on a magnetic stirrer (300 rpm) until the oleogelator melted. The liquid OG was then placed in the refrigerator at 4 °C for solidification and was further used to formulate the waffles.

#### Waffle formulation

Prior to obtaining the waffles, experiments were conducted to determine an optimal recipe, and the results were discussed somewhere else. For the purpose of the present study, an experimental design (DesignExpert; S tat‐Ease, Inc., Minneapolis, MN, USA) was used to provide a two‐level, three‐factorial design (Table 1), meaning that three factors were varied on two different levels to determine whether there are any influences on the waffles' properties. The factors that were varied were the whipping time of AF, its dry matter content and the fat type that was used in the recipe, oil or OG.

**Table 1:** The experimental design of the study

Sample	Whipping time (min)	AF concentration (g kg^−1^)	Presence of OG
1 (AF14‐3'U)	3	140	No
2 (AF14‐9'O)	9	140	Yes
3 (AF7‐9'O)	9	70	Yes
4 (AF7‐9'U)	9	70	No
5 (AF14‐9'U)	9	140	No
6 (AF7‐3'O)	3	70	Yes
7 (AF14‐3'O)	3	140	Yes
8 (AF7‐3'U)	3	70	No

Figure 1 illustrates the production process of the waffles. First, 40 g of AF was foamed until the time specified in the experimental design, with 15 g of sugar. In the last 30 s of foaming, 5 g of OG or oil (as specified for each sample) was added. In the resulting foam, 40 g of OF was added progressively until a dough was obtained, which was further cooked in a waffle maker (HB‐3562AB; 1300 W; Hausberg, Ergene, Turkiye) at 180–200 °C for 10 min. To prevent the sticking of the dough on the waffle maker's surface, cooking olive oil spray was used. After cooling, samples were analysed in terms of colour and texture, and they were kept in polyethylene bags in the refrigerator at 4 °C until the next day, when the remaining analyses were conducted. The doughs were also analysed for their textural parameters.

#### Texture analysis

Samples were analysed using the CT3 Brookfield Texture Analyzer (Brookfield Engineering Labs, Middleboro, MA, USA) equipped with a 10‐kg load cell. A textural profile analysis (TPA) test was performed for both the dough and the waffles. First, the dough was given a spherical shape (diameter 20 mm) and was analysed using the cylindrical probe TA11/1000 (diameter 25.4 mm; length 35 mm). Waffles were cut into rectangular shapes (30 × 25 × 15 mm) and were analysed using the cylindrical probe TA4/1000 (diameter 38.1 mm; length 20 mm). In all cases the test speed was set a 1 mm s^−1^ and the trigger load at 0.05 N. Results were computed using Texture Pro CT V1.6 (Brookfield Engineering Labs, Middleboro, MA, USA).

#### Chemical composition

An amount comprising 300 g kg^−1^ of sample was dried in the oven at 103 ± 2 °C until constant weight was achieved, and the moisture content was represented by the difference between the samples' water content before and after drying. To determine the ash content, 200 g kg^−1^ of the sample were placed in the calcination oven (B150; Nabertherm, Lilienthal, Germany) at 550 °C for 6 h until the ash obtained had a white colour without any traces of dark spots present. The Kjeldahl method was used to determine the crude protein content.

#### Instrumental colour determination

To assess the colour of the waffles, a portable colorimeter NR200 (3NH, Shenzhen, China) was used and the L*a*b* colour parameters were recorded, where L* represents the lightness of the samples, *a*+ represents red nuances, *a*– represents green, *b*+ represents yellow and b– represents blue (Ferreira *et al*., 2020). To obtain the actual colour of the samples, the aforementioned parameters were used to determine the HEX code of each sample. The strength of the colour is proportional to the chroma (C*), for which values indicate the degree of the colour saturation (Szymanowska *et al*., 2021) and this was calculated using:

(2) $$C^{*}=\sqrt{{a^{*}}^{2}+{b^{*}}^{2}}$$

#### Sensory analysis

The sensory evaluation was conducted with 13 inexperienced (i.e. untrained) participants who performed a seven‐point scale hedonic test and a preference test, aiming to investigate their acceptance of the product. The average age of the participants was 21.69 ± 1.03 years. Out of the 13 participants, 10 were female and three were male (all students) and none of them had gluten intolerance and none were vegan. Moreover, only two of them were not waffle consumers. Four samples were provided, but one of them, containing water instead of AF, was not considered for discussion because it did not fully represent a negative control for AF. One sample was chosen out of the eight of the experimental design based on the correlations found after performing a principal component analysis test. Along with this, two other control samples were used, containing either wheat flour or egg white (Table 2). Samples were evaluated together and were presented to the participants on plastic plates. They were cut in small rectangular pieces and identified using a random three‐letter code. Still water was also provided to the respondents becaus, after each sample, they were asked to clean their palates. First, the demographic data (age, gender, whether they were vegan, have a gluten intolerance and consume waffles) of the participants were collected, followed by the hedonic test which involved scoring the samples in terms of aspect, colour, texture, taste and general appreciation (in this order) Finally, participants were asked which sample was the one they preferred the most.

**Table 2:** The formulation of the samples used for the sensory analysis

Ingredient (g)	Wheat flour	Oat flour	Egg white	Aquafaba	Sugar	Oleogel
Waffle formulation						
WhF	40	–	–	40	15	5
6	–	40	–	40	15	5
EgW	–	40	40	–	15	5

### Statistical analysis

The analyses were performed in triplicate, except for the whipping time (one repetition) and rheology, foam stability and chemical composition (duplicate). Three‐way ANOVA was performed to see the influence of the factors, and their interaction, on the responses. One‐way ANOVA was conducted along with the Tukey's comparison test to determine whether there were statistically significant differences between samples and, if so, where those differences occurred, respectively. The significance level was defined at α = 0.05. A principal component analysis (PCA) test and a Pearson correlation test (95% confidence interval) were performed to choose one sample from the experimental design to be used in the sensory tests. Data were interpreted using Minitab 19.1 (Minitab, LLC, State College, PA, usa) and OriginPro 2024 (OriginLab Corp.).

## RESULTS AND DISCUSSION

### Results for AF

#### Refractive index

AF was found to have approximately 70 g kg^−1^ (AF7) dry matter content, which is consistent with other studies that have investigated this aspect.^9^ AF chemical composition is influenced by the processing parameters of the chickpea beans, such as boiling temperature, time and chickpea to water ratio,^18^ while its main components were reported to be carbohydrates, fibres and proteins.^19^ The concentrated AF samples had a dry matter content of approximately 140 g kg^−1^ (AF14) and 210 g kg^−1^ (AF21), and their potential to obtain foams, and later waffles, was investigated to observe the influence over the quality of the products.

#### FTIR

**Figure 2:**
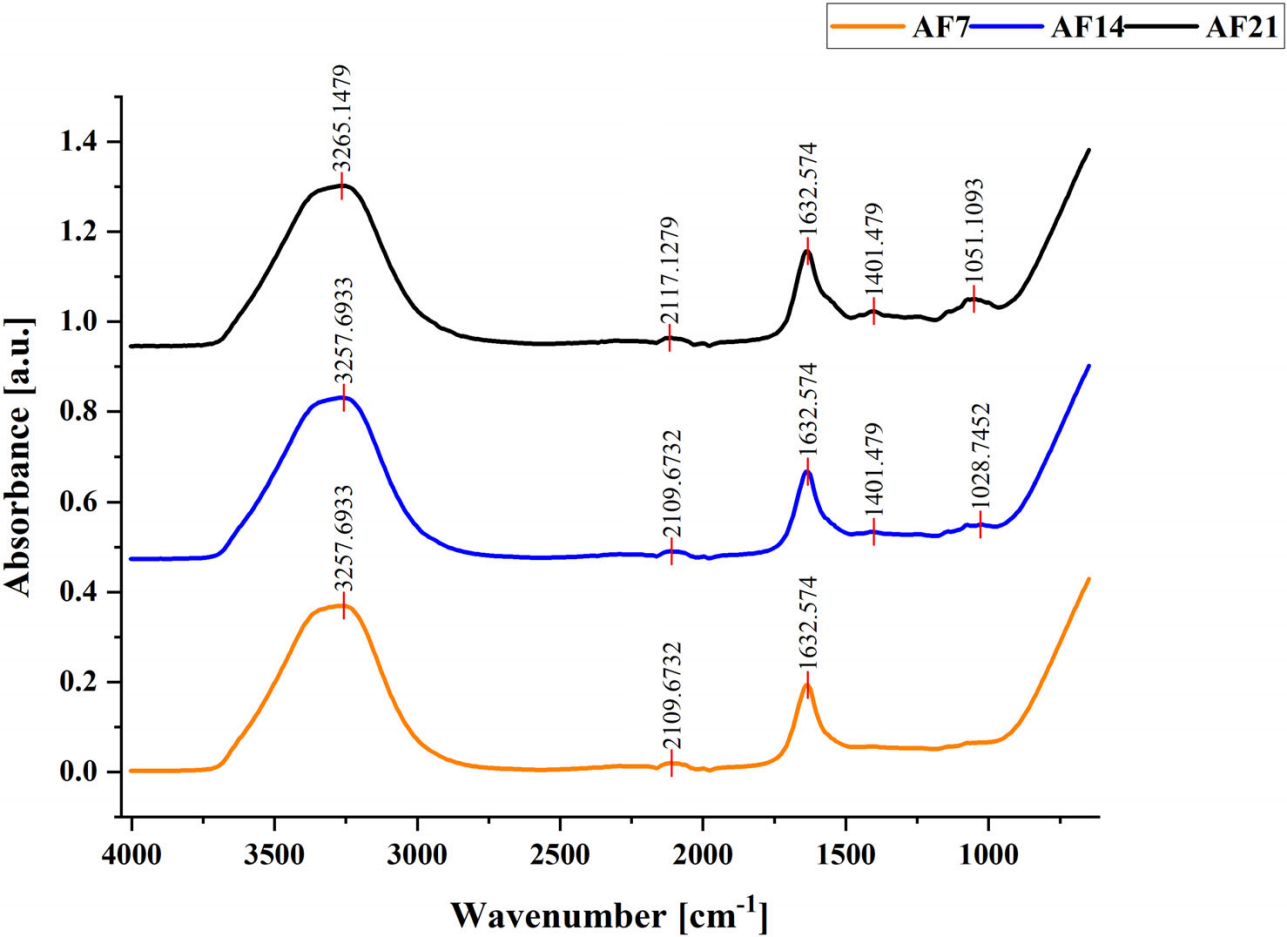
FTIR spectra of aquafaba. AF7 Aquafaba with 70 g kg−1 dry matter content, AF14 – 140 g kg−1 and AF21 – 210 g kg−1 dry matter content.

FTIR spectra of the three concentrations of AF are shown in Fig. 2. FTIR is a rapid method to detect protein fingerprints and can provide information regarding their molecular structure and composition as related to their functional groups and secondary structures.^18^ As observed in Fig. 2, three main classes were identified at different wavenumbers: 3570–3200 cm^−1^ (specific for hydroxy groups, thus indicating the presence of water), 2140–2100 cm^−1^ (terminal alkynes; C≡C bonds) and 1650–1590 cm^−1^ (primary amines; NH bends).^20^

The signals intensified as the AF concentration increased and additional peaks were recorded for AF14 and AF21 at 1401 cm^−1^ and within the range 1090–1020 cm^−1^ (specific to CN stretches of primary amines). Peak intensity variations and wavenumber shifts might be caused by the thermal treatment that AF underwent, thus changing the structural reorganisation of the proteins upon denaturation.^21^

It was previously found that AF contains mainly low molecular weight protein species, mostly albumins, which are known for their foaming properties.^13^ Proteins adsorb at the air–liquid interface after air bubbles are incorporated upon whipping, thus stabilising the foam.

#### Rheology

**Figure 3:**
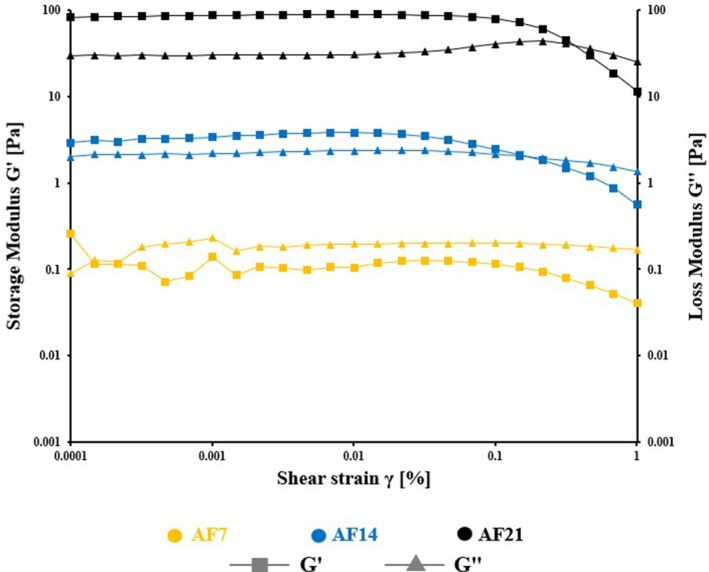
Amplitude sweep for AF7, AF14 and AF21. AF7 Aquafaba with 70 g kg−1 dry matter content, AF14 – 140 g kg−1 and AF21 – 210 g kg−1 dry matter content.

The amplitude sweep test (Fig. 3) was conducted to observe the linear viscoelastic region (LVR) of the samples, and the viscoelastic moduli, the storage modulus (*G*′) and the loss modulus (*G*′′), were recorded. According to the moduli, AF14 and AF21 exhibited a gel‐like texture (*G*′ > *G*′′), whereas AF7 showed a fluid behaviour (*G*′ < *G*′′). *G*′′ values were in the range 0.25–82.25 Pa and *G*′′ values were in the range 0.08–29.79 Pa. AF21 showed significantly higher values (*P* < 0.05) for both moduli within the LVR at a shear strain of 0.01 (*G*′ = 89.22 ± 3.17 Pa and *G*′′ = 30.67 ± 2.54 Pa) indicating a stronger gel as compared to AF14 (*G*′ = 3.81 ± 0.3 Pa and *G*′′ = 2.36 ± 0.2 Pa) which was softer. LVR is maintained until a crossing point between the moduli is reached (*G*′ = *G*′′) and, after that point, the structure of the gel is irreversibly destroyed. In this case, the overlapping occurred at a shear strain of 0.217% for AF14 and 0.466 for AF21, the latter gel being significantly stronger, finding also supported by the larger gap observed between *G*′ and *G*′′^22^ for this sample. The differences among samples might have been caused by the thermal treatment and the water content of the samples. AF7 was analysed as such after being collected from the cans, thus the higher water content made it more fluid. On the other hand, AF14 and AF21 were thermally treated to remove a part of the water, whereas, additionally, the proteins were denatured, thus causing them to exhibit the gel like behaviour that was observed.

#### Analyses of AF foams

As shown in Table 3, the whipping time decreased as the AF concentration increased, indicating that AF with a higher dry matter content needs less time to incorporate the maximum amount of air bubbles (until the overrun occurs)^19^ ultimately leading to the formation of the foam. For example, AF14 was whipped in 19 min. compared to AF21 that required only 14 min. Moreover, the foaming capacity, defined as the maximum amount of air incorporated into the foam, was significantly different among all samples. The foaming capacity of AF21 (82.91 ± 0.09%) was significantly higher compared to the other two samples, which had a foaming capacity of 80.29 ± 0.48% for AF7 and 76.79 ± 0.11% for AF14. Appearance of foams is presented in Figure 1s. Additionally, AF14 showed a significantly lower value compared to AF7, thus indicating that a doubling of the dry matter content still led to a foam with a lower foaming capacity. This aspect is rather interesting because proteins are the main components within the samples that are responsible for their foaming properties by creating a layer around the air bubbles as a result of the protein–protein interactions, as well as their interaction with other compounds.^18^ Consequently, proteins adsorb themselves at the air–water interface because of their amphiphilic properties and are thus able to maintain the structure of the foams.^23^ Increased temperatures of up to 100 °C might have an impact on the foaming properties of AF because such increased temperatures could potentially lead to complete protein denaturation, resulting in their aggregation, which will reduce their foaming properties.^18^ In the present study, AF of the highest concentration had the highest foaming capacity, although this pattern was not linear among samples because AF of the lowest concentration had the second highest foaming capacity. Serum loss can be influenced by gravity, which causes the liquid to drain from the foam,^19^ and was significantly higher for AF14 (17.5 ± 1.56%), but, surprisingly, no significant difference was found between AF7 (0.2 ± 0.00%) and AF21 (4.0 ± 3.68%). This finding, correlated with the lowest foaming capacity observed for AF14, reinforces the idea that the thermal treatment for AF should be standardised prior to conducting the experiments. Also, a higher serum loss for the samples with increased dry matter content might presumably confirm the hypothesis that increased temperatures lead to protein aggregation, and consequently resulting in less interactions with the water molecules and increased liquid drainage.

**Table 3:** Whipping time, foaming capacity and serum loss of aquafaba

Sample	Whipping time (min) and height of foams (mm)	Foaming capacity (%)	Serum loss (%)
AF7	19 min; 77 mm	80.29 ± 0.48 a	0.2 ± 0.00 b
AF14	17 min; 72 mm	76.79 ± 0.11 b	17.5 ± 1.56 a
AF21	14 min; 84 mm	82.91 ± 0.09 c	4.0 ± 3.68 b

*Note*: Values are the mean ± SD. Means with different lowercase letters are significantly different (*P* < 0.05). Abbreviations: AF7, aquafaba of 70 g kg^−1^ dry matter content; AF14, aquafaba of 140 g kg^−1^ dry matter content; AF21 aquafaba of 210 g kg^−1^ dry matter content.

### Results for waffles

#### Waffle formulation and the experimental design of the study

According to the experimental design of the study, eight samples were obtained (Figure 2s) and codified as mentioned in Table 1. Waffles are typically made from a batter,^17^ but, in this case, a dough was obtained, possibly as a result of the low quantity of liquids in the mixture. The first factor which was varied was the whipping time, and, empirically, it was decided that AF should not be whipped until the optimal time because a residual quantity of water in the foam was observed to be beneficial for the flour incorporation into the dough. The next factor was the concentration of AF, which was chosen based on the textural analysis of preliminary samples. The main reason to exclude AF21 was that waffles obtained with it had a significantly lower cohesiveness; thus, they could not be used for further experimentation. The last factor was the use of OG or oil in the recipe, which was considered given that waffles are normally made with saturated fats, usually of animal origin (e.g. butter). In this case, butter was replaced with OG and the samples were compared with their oil‐containing counterparts to observe any structural differences among them. Glycerol monostearate was used to obtain the OG because it was previously reported that it can form a three‐dimensional crystal network at a relatively low concentration, with applications in various bakery and pastry products.^24^ Waffles were formulated using gluten‐free OF, which led to the formation of a dough rather than a batter, given its higher content of fibres. Adding that to the absence of gluten, these aspects might have influenced the water‐binding capacity of OF.

#### Textural parameters

Textural parameters were obtained upon conducting the TPA, and the results are presented in Table 4. The hardness and the adhesiveness of the dough are two important parameters, higher values decreasing its quality.^25^ Dough hardness differed among samples according to the lipid ingredient used in their formulation. Samples containing AF7 and OG had a significantly higher hardness at the same whipping time compared to their oil counterparts. For example, 10.78 ± 0.16 N for sample 6 *versus* 4.81 ± 0.34 N for 8. The same pattern was found among samples formulated with AF14, but, in this case, the differences were not significant. Dough adhesiveness also showed higher values for the OG samples than for the oil samples. However, the values were not significantly different among all samples. Significant differences were found, for instance, between samples 3 and 4, with 3.1 ± 0.1 mJ and 0.93 ± 0.15 mJ, respectively. This might be an indication that adhesiveness is influenced by the saturated fatty acids contained by the GM, which led to an increase in its values. Moreover, considering different AF concentrations for the same whipping time and the same type of fat, not all differences were significant, as observed for samples 1 and 8. Given these results, it was postulated that the main influence on the texture of the dough was exerted by the presence or the absence of the OG in the recipe, whereas AF concentration had little impact because the results were inconsistent among samples. The whipping time had no significant impact on the textural parameters (*P* < 0.05).

**Table 4:** Textural parameters of the dough and the waffles

Sample	Dough		Waffle		
	Hardness, (N)	Adhesiveness, (mJ)	Hardness, (N)	Springiness, (mm)	Chewiness, (mJ)
1 (AF14‐3'U)	2.83 ± 0.24 c	1.03 ± 0.06 c	47.87 ± 7.98 bc	1.99 ± 0.07 cde	13.53 ± 3.32 abc
2 (AF14‐9'O)	6.47 ± 0.29 c	1.17 ± 0.15 bc	68.28 ± 12.93 ab	1.72 ± 0.15 e	17.46 ± 1.06 ab
3 (AF7‐9'O)	8.60 ± 1.40 b	3.10 ± 0.10 a	23.61 ± 0.93 c	2.31 ± 0.09 bcd	6.46 ± 1.00 c
4 (AF7‐9'U)	2.81 ± 0.10 c	0.93 ± 0.15 c	42.40 ± 7.72 c	2.78 ± 0.32 ab	14.10 ± 2.94 abc
5 (AF14‐9'U)	3.59 ± 0.03 dc	0.60 ± 0.26 c	81.29 ± 16.70 a	1.76 ± 0.09 de	14.65 ± 5.44 abc
6 (AF7‐3'O)	10.78 ± 0.16 a	1.53 ± 0.61 bc	26.24 ± 8.19 c	2.38 ± 0.24 abc	9.73 ± 4.35 bc
7 (AF14‐3'O)	2.89 ± 0.45 c	2.10 ± 0.40 b	45.5 ± 10.71 bc	1.97 ± 0.12 cde	15.53 ± 1.15 ab
8 (AF7‐3'U)	4.81 ± 0.34 d	1.00 ± 0.53 c	46.75 ± 2.35 bc	2.84 ± 0.15 a	18.05 ± 2.15 a

*Note*: Values are the mean ± SD. Means with different lowercase letters are significantly different (*P* < 0.05).

Regarding the texture of the waffles, the observed pattern was that samples with OG had lower values for hardness compared to waffles containing oil (e.g., 47.87 ± 7.98 N for 1 and 45.5 ± 10.71 N for 7) at the same AF concentration, although the differences were not significant (*P* > 0.05). The explanation might be that the saturated fatty acid (i.e. stearic acid) present in the GM leads to a softer texture.^26^ Sample 5 was significantly harder than all the other samples (81.29 ± 16.70 N), except for sample 2 (68.28 ± 12.93 N). Springiness was significantly lower for waffles with higher concentration of AF and the same fat contained, except for samples 6 (2.38 ± 0.24 mm) and 7 (1.97 ± 0.12 mm), which did not differ significantly. Nevertheless, all values tend to be rather low for such products, and, as already reported by Amran and Mohamad,^27^ this is because fibre and the absence of gluten reduce the elasticity of the dough, and the resulting product will be denser. Regarding chewiness, sample 3 showed the lowest value: 6.46 ± 1.00 mJ. Chewiness is influenced by cohesiveness which might have been reduced as a consequence of using gluten‐free OF, which lacks the two proteins, gliadin and glutenin, that lead to the formation of a three‐dimensional network giving the products a stable and cohesive structure.^26^

#### Chemical composition

The chemical composition of waffles is shown in Table 5.

**Table 5:** The chemical composition and chroma of waffles

Sample	Moisture (g kg^−1^)	Ash (g kg^−1^)	Protein (g kg^−1^)	Chroma
1 (AF143'U)	199.0 ± 1.65 ab	16.4 ± 0.09 b	88.1 ± 0.88 ab	35.00 ± 3.83 b
2 (AF149'O)	192.1 ± 0.10 bc	15.6 ± 0.06 b	87.7 ± 0.34 ab	38.12 ± 4.37 ab
3 (AF79'O)	249.0 ± 2.85 a	12.1 ± 0.08 a	78.2 ± 0.39 ab	37.11 ± 0.26 ab
4 (AF79'U)	211.3 ± 0.19 ab	12.2 ± 0.02 a	76.5 ± 0.09 ab	36.50 ± 2.41 ab
5 (AF149'U)	150.0 ± 0.58 c	16.1 ± 0.02 b	92.3 ± 0.25 a	42.80 ± 0.37 a
6 (AF73'O)	187.4 ± 0.07 bc	12.0 ± 0.17 a	74.2 ± 0.06 b	31.09 ± 0.62 b
7 (AF143'O)	187.7 ± 1.09 bc	15.5 ± 0.02 b	90.4 ± 0.49 ab	34.80 ± 1.50 b
8 (AF73'U)	192.5 ± 0.16 bc	11.7 ± 0.01 a	76.3 ± 0.20 ab	37.71 ± 4.19 ab

*Note*: Values are the mean ± SD. Means with different lowercase letters are significantly different (*P* < 0.05).

The moisture content varied inconsistently among samples, aspect that was possibly influenced by the cooking process of the waffles. However, interestingly, the moisture values ranged between 150.0 ± 0.58 g kg^−1^ and 249.0 ± 2.85 g kg^−1^. This aspect could be problematic because it will increase their susceptibility to spoilage; normally, waffles should have a moisture content of maximum 140.0 g kg^−1^.^17^ The explanation could be that OF made it more difficult for the water to be removed from the cooking waffles because of the increased water‐binding capacity of OF caused by its fibre content, which has more hydroxy groups, thus leading to more interactions with the water molecules via hydrogen bonds.^28^

Ash content was significantly different among samples with different AF concentrations. AF14 had a significantly higher ash content when compared to AF7 samples (e.g. 12.20 ± 0.02 g kg^−1^ for sample 4 and 16.10 ± 0.02 g kg^−1^ for sample 5). Additionally, among samples with the same dry matter content for AF, no significant differences were observed. Therefore, AF significantly increased the ash content, as expected, because it was the only ingredient for which the composition varied.

The protein content did not differ significantly among samples, but it was observed that those containing AF14 registered higher values. The only significant difference was found between sample 5, which had a higher protein content compared to sample 6; namely, 92.30 ± 0.25 g kg^−1^ *versus* 74.20 ± 0.06 g kg^−1^, respectively.

Most of both ash and protein content came from the oat flour, which was quantitatively consistent for all samples, and so the variations recorded are most probably a result of the different dry matter content of AF. This result is plausible because the normal protein concentration of AF, as previously determined in another study is 260 g kg^−1^ as per its dry matter content^9^; thus, doubling the concentration of AF will also increase its protein and ash contents.

#### Colour parameters

**Figure 4:**
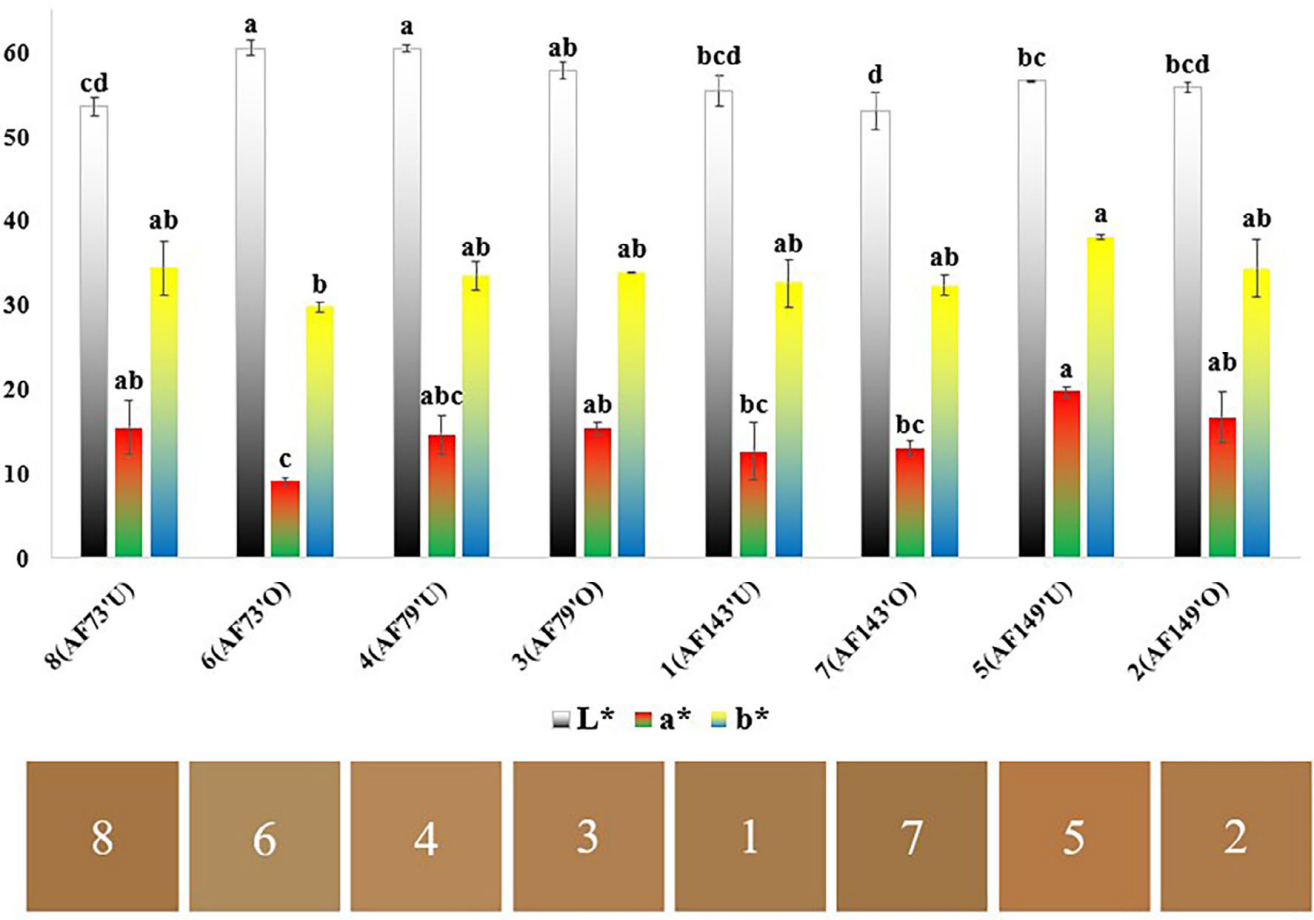
L*a*b* colour parameters of the waffles and their actual colour. Means with different lowercase letters are significantly different (*P* < 0.05).

The colorimetric three‐dimensional coordinates and the actual colour of samples, as determined using their HEX code, are shown in Fig. 4.

As can be seen, L* values varied inconsistently among samples, with significant differences being observed, for example, between samples 6 (60.5 ± 0.95) and 7 (53.03 ± 2.19), with the former having a greater brightness. A higher chroma value means a a higher colour intensity and, as shown in Table 5, not all differences were significant among samples for this parameter. For example, sample 7 (34.8 ± 1.5) had a significantly lower colour intensity compared to sample 5 (42.8 ± 0.37), but it did not differ significantly when compared to sample 6 (31.09 ± 0.62). Because the colour of waffles is mainly given by the Maillard reactions between reducing sugars and proteins,^29^ it was expected to observe more pronounced differences between samples with AF7 and those with AF14, but this did not occur. Moreover, the fat used did not affect the colour of samples, whereas the whipping time had an influence on the a*, b* and chroma values (*P* < 0.05). These findings suggest that, in the case of the colorimetric properties of the waffles, differences in terms of the dry matter content of AF between samples were negligible, and consequently did not influence the colour of the samples.

#### PCA and sensory analysis

**Figure 5:**
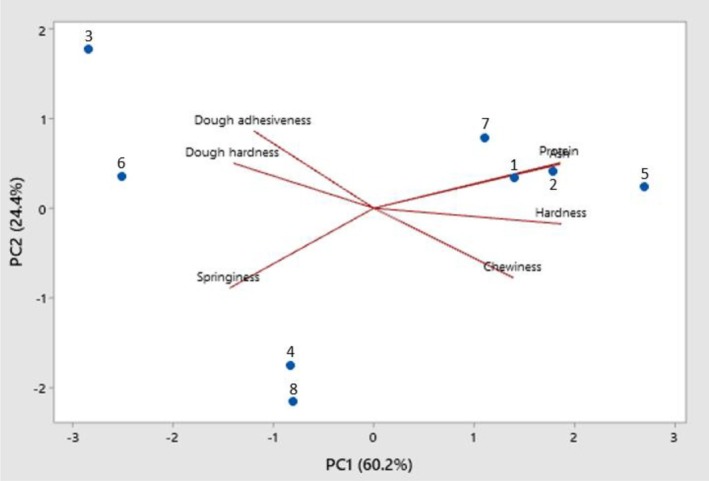
Bi‐plot of dough hardness, dough adhesiveness, protein content (waffles), ash content (waffles), hardness (waffles), springiness (waffles) and chewiness (waffles).

First, the PCA was conducted to choose a sample for the sensory evaluation. In this regard, some physicochemical attributes were considered, namely dough hardness and dough adhesiveness, and the following waffles' attributes: protein, ash, hardness, springiness, and chewiness. The biplot can be observed in Fig. 5.

Principal component (PC) 1 explained 60.2% of the variance and PC 2 explained 24.4% of the variance.

The pairwise Pearson correlation investigated the correlations among all analysed attributes, and significant correlations (*P* < 0.05) were found. A high positive correlation (*r* = 0.743) was found between protein and hardness. Springiness was highly negatively correlated with both protein (*r* = −868) and ash (*r* = −881). Finally, a strong positive correlation between ash and protein (*r* = 0.962) was also observed. Considering these results, a higher protein content led to an increased hardness and reduced springiness of the waffles, whereas increasing the protein content also increased the ash content. This aspect was already observed when comparing AF7 with AF14, with the latter having a higher protein content, although the increase was not significant, at the same time as showing a significantly higher ash content. Therefore, based on the significant correlations that were found, it was decided to choose a sample with AF7, such that it will have a lower protein and ash content, and, to improve the time efficiency, it was also decided to whip AF for only 3 min because this parameter did not to impact the texture. Additionally, the sample needed to contain OG because the aim was to investigate its feasibility as an alternative ingredient for saturated fats in waffles. Consequently, sample W6 was used in the consumers tests along with control samples for the oat flour, which was replaced with all‐purpose wheat flour (WhF), and for AF, which was as replaced with egg white (EgW). The complete formulation of the samples is shown in Table 2 and the hedonic scores are shown in Table 6.

**Table 6:** Hedonic test results of analysed waffles

Attribute	WhF	W6	EgW
Aspect	5.38 ± 1.60 a	4.38 ± 1.75 a	4.38 ± 1.38 a
Colour	5.38 ± 1.60 a	4.61 ± 1.71 a	4.69 ± 1.65 a
Texture	4.84 ± 1.46 a	4.15 ± 1.90 a	3.84 ± 1.62 a
Taste	4.92 ± 1.65 a	4.38 ± 1.85 a	4.00 ± 1.50 a
General appreciation	4.84 ± 1.57 a	4.69 ± 1.60 a	4.30 ± 1.65 a

*Note*: Values are the mean ± SD. Means with different lowercase letters are significantly different (*P* < 0.05). Abbreviations: WhF, sample with wheat flour; W6, experimental sample; EgW, sample with egg white.

As can be seen, rather medium hedonic scores were registered for all samples, indicating a slight appreciation from consumers. WhF had the highest scores for all attributes compared to W6 and EgW, although the differences were not significant. Regarding the preference test, the most preferred sample was WhF, with 58% of respondents choosing it, followed by W6 (25%) and EgW (17%). The consumers' preference might have been influenced by their perception of the products' texture, given that, in the hedonic test, this attribute is directly proportional to consumers' preference (4.84 ± 1.46, 4.15 ± 1.90 and 3.84 ± 1.62, respectively). Moreover, this finding was supported by a strong positive correlation found between preference and texture as a sensory attribute (*r* = 0.971), but it was not significant (*P* = 0.154). Given that WhF was the most appreciated and preferred sample, this might indicate that the main ingredient leading to lower scores for samples W6 and EgW was OF, highlighting the importance of conducting the evaluation with coeliacs.

## CONCLUSIONS

In the present study, the potential of AF as a foaming ingredient in the manufacturing of waffles was revealed, with the use of oleogel as a saturated fat replacer in this product also being feasible. Regarding AF from chickpea cans, it was found to have a 70 g kg^−1^ dry matter content, which was then doubled and tripled by boiling to explore its properties. FTIR analysis revealed the protein specific peaks (for which the intensity increased with the dry matter content of AF) that are responsible for its foaming properties. AF7 had a liquid behaviour, whereas AF14 and AF21 exhibited a gel‐like texture; the gel was stiffer and stronger as the dry matter content increased, indicating that the thermal treatment affected the structure of the proteins and consequently their gelling/whipping properties. The whipping time decreased as the AF concentration increased, but foaming capacity and serum loss varied inconsistently among samples, possibly as a result of the thermal treatment. To obtain the waffles, three factors were varied on two levels in a three‐factorial, two‐level design: AF concentration and whipping duration, as well as the usage of oil and oleogel. The dough of waffles with AF7 and OG had a significantly higher hardness at the same whipping time compared to their oil counterparts. Dough adhesiveness also had higher values for the OG samples compared to oil, although not all differences were significant in this case. Dough texture was impacted by the type of fat used in the recipe. The hardness of waffles did not differ significantly for samples with the same AF dry matter content that contained different types of fat. Springiness was rather low for such products, possibly as a result of the oat flour and the absence of gluten, which also affected chewiness by making the waffles less cohesive. The moisture content was slightly high for this type of products, most probably as a result of the OF's fibres, and could increase their susceptibility against spoilage. The ash content of waffles increased significantly with AF concentration, whereas protein content also increased, although the differences were not statistically significant. The whipping time did not influence the textural parameters of the products, but it had an influence on the colour. Significant correlations were found between protein and hardness, springiness and ash, and ash and chewiness. The waffle containing wheat flour was appreciated the most in the hedonic test, suggesting that gluten‐free oat flour is not readily accepted by all consumers, especially those who are not gluten intolerant. AF, a sustainable ingredient, can be employed in manufacturing waffles, while concentrating AF could improve the chemical composition of waffles (ash and protein content), which were correlated with the textural attributes.

## AUTHOR CONTRIBUTIONS

CS was responsible for writing the original draft and investigations. AP was responsible for reviewing and editing, supervision and project administration. AP and AEM were responsible for resources. AEM was responsible for formal analysis and resources. VM was responsible for validation, methodology and supervision.

## FUNDING INFORMATION

This work was supported by a grant from the Romanian Ministry of Research, Innovation and Digitization project number PN‐IV‐P2‐2.1‐TE‐2023‐1206 within PNCDI IV.

## Supporting information


**Data S1.** Supporting information.
**Figure S1.** The foams obtained using aquafaba of different dry matter contents: AF7 — 70 g kg^−1^ dry matter content, AF14 — 140 g kg^−1^ and AF21 — 210 g kg^−1^ dry matter content.
**Figure S2.** The samples of waffles: 1 — AF14‐3′U; 2 — AF14‐9′O; 3 — AF7‐9′O; 4 — AF7‐9′U; 5 — AF14‐9′U; 6 — AF7‐3′O; 7 — AF14‐3′O; 8 — AF7‐3′U.

## Data Availability

The data that support the findings of this study are available from the corresponding author upon reasonable request.
