# Supplementary material for: Designing nutritionally improved waffles containing glycerol monostearate based oleogel and aquafaba
Source: J Sci Food Agric. 2026 May 15;106(11):6779–88. doi: 10.1002/jsfa.70717 (PMC13341060; doi:10.1002/jsfa.70717)
Supplement: Supplementary file 1 — Data S1. Supporting information. Figure S1. The foams obtained using aquafaba of different dry matter contents: AF7 — 70 g kg−1 dry matter content, AF14 — 140 g kg−1 and AF21 — 210 g kg−1 dry matter content. Figure S2. The samples of waffles: 1 — AF14‐3′U; 2 — AF14‐9′O; 3 — AF7‐9′O; 4 — AF7‐9′U; 5 — AF14‐9′U; 6 — AF7‐3′O; 7 — AF14‐3′O; 8 — AF7‐3′U. [file JSFA-106-6779-s001.docx]

**Supplementary material**


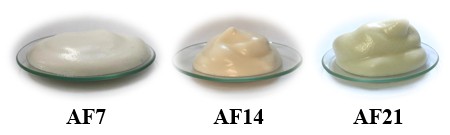


Figure S1. The foams obtained using aquafaba of different dry matter contents: AF7 – 70 g kg^−1^ dry matter content, AF14 – 140 g kg^−1^ and AF21 – 210 g kg^−1^ dry matter content.


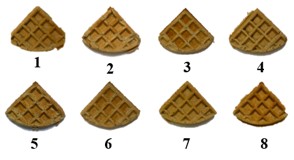


Figure S2. The samples of waffles: 1 - AF14‐3'U; 2 - AF14‐9'O; 3 - AF7‐9'O; 4 - AF7‐9'U; 5 - AF14‐9'U; 6 - AF7‐3'O; 7 - AF14‐3'O; 8 - AF7‐3'U.
